# The Campbell Paradigm as a Behavior-Predictive Reinterpretation of the Classical Tripartite Model of Attitudes

**DOI:** 10.1027/1016-9040/a000364

**Published:** 2019-07-10

**Authors:** Florian G. Kaiser, Mark Wilson

**Affiliations:** ^1^ Institute of Psychology, Otto-von-Guericke University Magdeburg, Germany; ^2^ Graduate School of Education, University of California, Berkeley, CA, USA

**Keywords:** attitudes, attitude-behavior consistency, attitude measurement, tripartite model, Campbell Paradigm

## Abstract

**Abstract.** In this article, we introduce the “Campbell Paradigm” as a novel variant of Rosenberg and Hovland’s (1960) tripartite model of attitudes. The Campbell Paradigm is based on a highly restricted measurement model that speaks of a compensatory relation between a person’s latent attitude and the costs that come with any specific behavior. It overcomes the overarching weakness of the original tripartite model (i.e., its relative irrelevance for actual behavior) and offers a parsimonious explanation for behavior. Even though this seems attractive, we also discuss why the paradigm has not gained momentum in the 50 years since it was originally proposed by Donald T. Campbell. To demonstrate the paradigm’s suitability even when implemented with an unrefined instrument in a domain where it has not been used previously, we apply the paradigm to a classic data example from attitude research from the 1984 US presidential election to account for the electorate’s voting intentions and actual voting behaviors.

According to Baumeister, Vohs, and Funder (2002), the science of behavior is progressively missing its target because “… studies on [actual] behavior are dwindling rapidly…” (p. 396). The situation is not any different in attitude research, which comprises the third main cluster of mental (i.e., inherently psychological) constructs after intellectual abilities and personality traits. In attitude research, behavior-explanation models (e.g., the theory of planned behavior; Ajzen & Fishbein, 2005) dominate much of contemporary behavioral research. In these behavior-explanation models, “… attitude … is … [typically] one of many factors that influence behavior” (Ajzen & Fishbein, 1980, p. 26). However, using such multifactorial models to explain behavior implies only a *fractional*, often *feeble* (and, at most, *inconsistent*) attitude-behavior relation. The sound measurement of attitudes, by contrast, demands a *substantial* attitude-behavior relation because attitude measurement entirely and unconditionally rests upon the link between an attitude and some manifestations (i.e., observable behavior).

The most common measurement model of (explicit) attitudes is still the venerable tripartite model of attitudes by Rosenberg and Hovland (1960; see also, e.g., Eagly & Chaiken, 1993). This tripartite model is a latent variable model that describes the relation between a latent attitude and its cognitive, affective, and behavioral manifestations (see Figure 1A). Next to its use as a measurement model for individual attitudes, it also logically represents an account of attitude-relevant individual behavior. In other words, the very model used to establish an estimation of a latent attitude also represents a behavior-explanation model because it is intended to account for any kind of manifestation of an attitude (e.g., verbal responses in questionnaires, facial expressions, and actual behavior).

**Figure 1:**
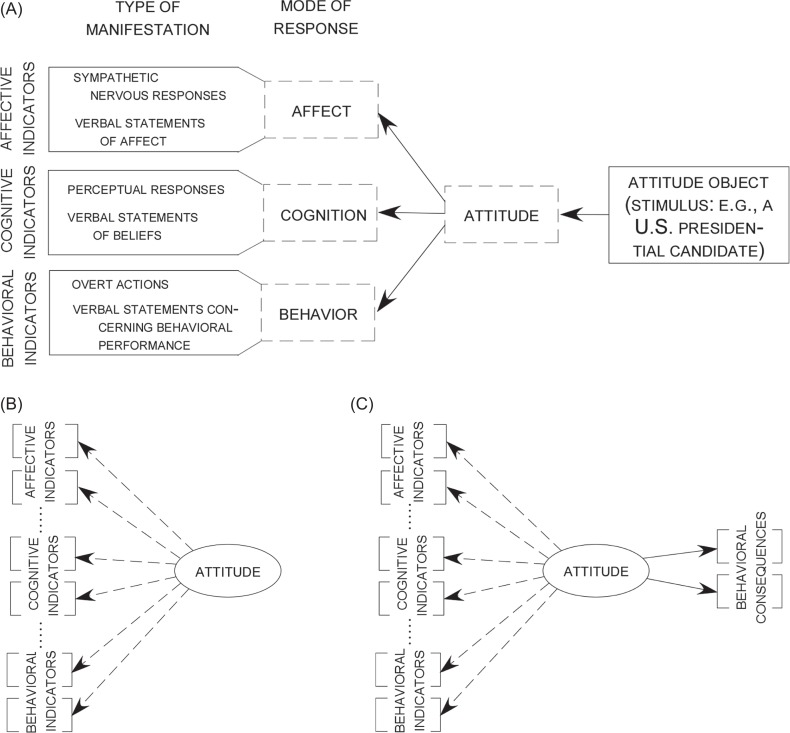
Different versions of the tripartite model of attitudes according to Rosenberg and Hovland (1960). (A) Schematic model; (B) Reflective measurement model. The attitude represents the only latent variable, whereas its manifestations fall into three distinguishable classes of responses. (C) A reflective measurement model and a behavior-explanation model combined.

Due to its limited relevance for overt behavior, scholars have challenged the tripartite model as being problematic and have proposed alternative measurement models of individual attitudes (for overviews, see, e.g., Krosnick, Judd, & Wittenbrink, 2005; Schwarz, 2008) – often without corroborating the behavioral relevance of their alternative propositions (see, e.g., Dalege et al., 2016).

As a scientific community, we should avoid perpetually reinventing the wheel and with it fostering the degradation of our methodological acuity. As an alternative, we offer a promising variant of the venerable tripartite measurement model. This variant is called the Campbell Paradigm; it is named after Donald T. Campbell (1963), who had proposed the original measurement principle on which it was based. In this article and for the first time, we explain why the Campbell Paradigm can be viewed as a highly restricted variant of the classical tripartite measurement model. But not only can the Campbell Paradigm be applied to the measurement of attitudes, as we will demonstrate, it also allows one to parsimoniously explain and substantially predict behavior. As we will not elaborate on the Campbell Paradigm and its constituents in great detail here, we refer to Kaiser, Byrka, and Hartig (2010) for such a comprehensive account.

In the first part of our article, we will briefly summarize the classical version of the tripartite model of attitudes. Subsequently, we will describe the ways in which the Campbell Paradigm represents a variant of the tripartite model and explain why this paradigm has failed to gain momentum as a foundation for the science of behavior over the more than 50 years since it was suggested by Campbell. In the second part of the article, we will apply the paradigm to data from the 1984 US presidential election to account for the voting intentions and voting behavior of the electorate.

We chose these data for two reasons: first, because voting behavior is a classic example in attitude research (see, e.g., Fishbein & Ajzen, 1981; Fishbein & Coombs, 1974), and it offers an example that is distinct from all prior applications of the Campbell Paradigm, most of which have been about environmental protection (see e.g., Kaiser et al., 2010; Kaiser, Hartig, Brügger, & Duvier, 2013); second, because the very same dataset was recently used in a *Psychological Review* article in which Dalege et al. (2016) argued against the tripartite model and proposed a new unrestricted alternative measurement model. As we will demonstrate in our article, not only can the Campbell Paradigm be applied to Dalege et al.’s data, but it can also parsimoniously explain and substantially predict people’s voting behavior. By contrast, Dalege et al. did not even attempt to predict voting behavior with their own model. The technical details of our empirical test (e.g., descriptions of the sample and the data) can be found in the Appendix. Examples of attitude measures that more closely fit our variant of the tripartite model – including behavioral self-reports and various types of evaluative statements – can be found in Byrka and Kaiser (2013) and Kaiser, Merten, and Wetzel (2018).

## Measurement Models of Individual Attitudes

Before turning to the specifics of the Campbell Paradigm, we will summarize the generic ideas behind Rosenberg and Hovland’s (1960) tripartite measurement model of (explicit) attitudes of individuals.

### The Tripartite Model of Attitudes: A Latent Variable Measurement Model

The tripartite model of attitudes is a latent variable model that is based on the assumption that the latent variable (i.e., a person’s attitude) elicits three modes or types of manifestations: a person’s cognitive, affective, and behavioral responses to an attitude object (e.g., a US presidential candidate, or a Dutch soccer team, say PSV Eindhoven; see Figure 1A). The latent attitude is in turn expected to be *formed* by the attitude object.

To be objectively recognizable, a person’s *latent* attitude toward an attitude object (e.g., PSV Eindhoven) must be displayed as *manifest* behavior (including all sorts of corporal reactions). According to the tripartite model, these manifestations can take three generic forms: for example, when the person verbally states that PSV is the “best” team and will therefore win the championship (i.e., a cognitive response), when he/she physically attends a PSV game (i.e., a behavioral response), and when his/her face erupts into expressions of joy when PSV scores a goal (i.e., an affective response). Conceptually, in this model, the attitude (the latent variable) is linked with three types of manifestations (i.e., observable behavior; see Figure 1A).^1^ More specifically, the tripartite model can be applied to explain the occurrence of these manifestations according to the level of the latent attitude a person embodies.

When the three types of manifestations are regarded as attitude indicators, the tripartite model can be viewed as a measurement model for attitudes (see Rosenberg & Hovland, 1960). The distinction between indicators and responses is, however, arbitrary and merely a linguistic one. In Figure 1B, cognitive, affective, and behavioral manifestations are regarded as indicators of the latent attitude. To allude to causation between latent attitudes and their various manifestations, it is common to use arrows to link latent variables with their manifestations. Typically, such models are called *reflective* measurement models (e.g., Edwards & Bagozzi, 2000).

When the tripartite model is implemented as a measurement model, a person’s latent attitude toward, say, US presidential candidate X is typically derived from the respondent’s evaluative statements on questionnaires. As above, these evaluative reactions can be (a) verbal expressions of one’s cognitive valuation of the attitude object (e.g., candidate X sets a good example), (b) self-reports of one’s affective reactions (e.g., X makes me feel proud), (c) self-reports of one’s behavioral intentions (e.g., I will vote for X) or of one’s past behavior with respect to the attitude object (e.g., I voted for X), or any combination of these indicators. Note that the latent attitude can become manifest in many ways and formats, and it is not necessary to consider them all. Ideally, it should not matter which combinations of these indicators are used to measure the underlying *latent* variable (i.e., one’s attitude toward candidate X) that is believed to control the person’s *manifest* reactions (see, e.g., Eagly & Chaiken, 1993; Rosenberg & Hovland, 1960). Even the exclusive use of cognitive and affective indicators would be acceptable.

In the next section, we will describe the Campbell Paradigm, which we understand might be unfamiliar to some readers (for more details, see Kaiser et al., 2010). Therefore, we will describe it in comparatively a bit more detail than the detail we used for the tripartite model. In our description of the Campbell Paradigm, we refer to examples from the environmental-protection domain in which the paradigm was originally developed.

### The Campbell Paradigm

Donald T. Campbell (1963) proposed the original conceptual idea that the relative cost of the implementation (i.e., the difficulty) of a behavior is a decisive element for understanding the relation between a (latent) attitude and a (manifest) behavior.^2^ Accordingly and foremost, we can and should make use of the order (in terms of costs) of behavior in the measurement of attitudes.

For example, an environmentalist (i.e., a person who aspires to protect the environment and who, one might assume, holds a pronounced pro-environmental attitude) is typically expected to engage in a set of activities that reflect his or her attitude. For instance, she/he may publicly acknowledge that climate change is caused by humans, vote for representatives with a known pro-environmental record, recycle cardboard regularly, and eliminate foods that are particularly environmentally harmful (e.g., meat) from his/her diet. Generally, the person’s esteem for an attitudinal object (e.g., environmental protection) or goal (e.g., preserving the environment) becomes obvious in the extent to which he/she engages in more and more difficult behaviors that involve increasingly demanding barriers or progressively more painful sacrifices (i.e., behavioral costs). Thus, Campbell’s idea is that the *cost order* of behavior (i.e., reactions, indicators, or items) can and should be used as the basis for the measurement of individual attitudes.

#### A Measurement Model Grounded in Item Order

Consistent with his suggestion that the order of items should be seen as paramount to the measurement of attitudes, Campbell (1963) originally proposed the Guttman (1944) model as the optimum model for measuring attitudes. The Guttman model and its related scalogram approach have been widely used in the past in psychological and sociological studies involving domains that are seen as inherently ordered, such as in cognitive development (e.g., Cousins, Siegel, & Maxwell, 1983), cognitive decline (e.g., Tractenberg, Yumoto, Aisen, Kaye, & Mislevy, 2012), or involvement with drugs (e.g., Donovan & Jessor, 1983).

The Guttman model is based on the use of a person-invariant ordered set of items (*I*_1_*, I*_2_*,* … *I*_*k*_) to measure a latent variable (e.g., Wilson, 2013). This is similar to a math ability test on which students are asked to solve a set of increasingly demanding math problems (e.g., addition, multiplication, integration, etc.). In such a test, the presumption is that whether or not the students as a whole answer the items correctly reflects the difficulty of the items. Accordingly, item order means that the relative difficulty or costs of each behavioral indicator will be approximately the same across the population. A Guttman ordering can be summarized as thus: If a person affirms a demanding indicator (e.g., *I*_*n*_), then he or she will necessarily affirm all less demanding indicators (i.e., all *I*_*j*_ with *j* < *n*, where it is assumed that the items are ordered by difficulty) as well. Vice versa, if a person fails to affirm an undemanding indicator, he or she will not affirm more demanding ones.

Behavioral costs can appear to be rather small, such as when a person publicly expresses his or her unfavorable view of a candidate for the US presidency (e.g., Walter Mondale) by marking a box on a survey. Costs for other behaviors are obviously more substantial, such as when a person actively or financially supports a candidate’s campaign (e.g., Ronald Reagan, Mondale’s opponent in the 1984 election). Behavioral costs come in many different forms, for example, when a behavior involves personal effort, time, personal sacrifices, or money or when a behavior involves transgressions of social norms, expectations, or display rules. In a particular sociocultural context (e.g., a given society), these costs commonly apply to all people (see e.g., Kaiser & Keller, 2001; Scheuthle, Carabias-Hütter, & Kaiser, 2005), though this uniformity must be investigated in each particular context (and is typically part of any Rasch-model test).

On a Campbellian attitude measure, respondents are challenged by facing a set of increasingly demanding behavioral indicators, and individual attitudes are indicated by the maximum number of behavioral costs that a person is willing to surmount. In other words and according to Campbell’s proposal, a person’s esteem for an attitudinal object (e.g., a particular presidential candidate) or goal (e.g., the election of the particular candidate) becomes clear in the face of the behavioral costs the person is willing to endure in order to reveal positivity toward the object in question or to attain the related goal.

Campbell’s original proposition was successfully tested with the Guttman model (see Raden, 1977). Nevertheless, Campbell’s proposition was not picked up again until recently, perhaps due to the problems inherent to Guttman scaling, for which a single and perfect discrimination point is unrealistically assumed to exist between any two attitude levels (see, e.g., Kofsky, 1966, pp. 202–203), but also because of an apparent concern about conceptual circularity (see, e.g., Raden, 1977; see also Dawes & Smith, 1985; Greve, 2001).

#### Nontrivial Explanation of Behavioral Responses

Deriving an environmental attitude measure from the same behavioral indicators that are supposed to subsequently be explained by the attitude would confound the measure. In other words, if a person’s attitude is estimated through the behaviors that a person enacts, we cannot really be surprised to find the very *same* behaviors explained by this attitude on an empirical test. This is why Campbell’s proposal for measuring individual attitudes was initially regarded as circular, even by Campbell himself (e.g., Campbell, 1963), and has not been pursued as a model for explaining behavior. The solution to this conceptual conundrum is, as we will demonstrate, the logical and practical separation of the *indicators* (i.e., the manifestations used to estimate the individual level of an attitude) and the *consequences* of an attitude (e.g., its manifest *effects*, the criteria to be explained).

When Kaiser et al. (2010) saw reason to adopt Campbell’s (1963) original idea of using the costs of behavior as a decisive element in the measurement of attitudes, they replaced the Guttman model with the Rasch measurement model (for more details about the Rasch model, see Rasch, 1960/1980; for a recent account, see, e.g., Wilson, 2005). Note that the argument for interpreting the Rasch family of models as probabilistic Guttman models has been made several times in the literature, and hence, we will not make the case for them here (see, e.g., Wilson, 2013). Whereas the first part of Kaiser et al.’s (2010) proposal involves viewing the tripartite model as a reflective measurement model – see Figure 1B – the second part involves distinguishing indicators from consequences.

In contrast to common beliefs about measurement (see, e.g., De Houwer, Gawronski, & Barnes-Holmes, 2013), the behavioral *indicators* that are used to measure an attitude (e.g., verbal expressions of one’s cognitive and affective reactions to an attitude object) can be designed to be different from the behavioral *consequences* that are modeled as caused by an attitude (e.g., active behaviors, retrospective reports of one’s actual behavior or prospective expressions of one’s intention to act in a certain way; see Figure 1C). Note that in Figure 1C, the indicators are linked with attitudes by dashed arrows, whereas the consequences are linked by solid arrows. Thus, the pertinence of particular responses to an attitude (either as indicator or as consequence) can become empirically recognizable without circularity concerns.

Within the Campbell Paradigm, estimates of individual attitudes can be derived from any set of manifest indicators of an attitude (e.g., evaluative statements about, and affective reactions to the attitude object, verbal reports about behavioral intentions, or observations and self-reports of behavior). It would indeed constitute circular logic if the behavioral effects – the set of behaviors-to-be-explained – were included among the indicators that were used to estimate the attitude. However, when there is a logical or a practical (e.g., a temporal) separation between the indicators and the specific behavioral consequences of an attitude, then there is no logical fallacy. In other words, *measuring attitudes and explaining behavior on the basis of individual attitudes can be treated as two separate tasks* in the Campbell Paradigm (see, e.g., Byrka, Kaiser, & Olko, 2017; Kaiser & Byrka, 2015; Taube, Kibbe, Vetter, Adler, & Kaiser, 2018).

Of course, this logical or practical distinction must be examined in each instance in which the paradigm is used. In the case of the presidential election data, we will see that people’s expressions of their intentions to vote for a certain candidate and their post-election self-reports of voting behavior are both – at least, logically – distinct from the same people’s manifest cognitive and affective indicators of their attitudes. Whereas intentions were, in this case, only logically distinct – by means of item wording – from the cognitive and affective indicators, the behavioral self-reports were both logically and temporally distinct from the cognitive and affective indicators of the attitude measure.

#### The Rasch Model as a Feasible Alternative

Whereas Campbell (1963) originally proposed the deterministic Guttman model as appropriate for attitude measurement, Kaiser et al. (2010) viewed the Guttman model as unrealistically stringent because it is based on the assumption that there is no measurement error in the system. In concert with other researchers (e.g., Kofsky, 1966; Wilson, 2011, 2013), Kaiser et al. proposed the probabilistic models as a solution and the Rasch model as a good way to model individual attitudes (for similar reasoning in the domain of intellectual abilities, see Wilson, 2005).

In contrast to the Guttman model, the Rasch model leaves room for observational irregularities (i.e., measurement error) because, rather than directly modeling a person’s actual engagement in a specific behavior (including verbal behavior), it models the person’s *probability* of engaging in the behavior. In other words, the Rasch model reduces the goal of applying the measurement model from predicting people’s engagement in a behavior to predicting the probability of engaging in a behavior (e.g., verbal behavior on surveys).

For Kaiser et al.’s (2010) formulation of the Campbell Paradigm for attitude research, it nevertheless remains an essential commitment to the paradigm to establish the order of the indicators of an attitude. In the Campbell Paradigm, however, it is not essential for the indicators from which an attitude is inferred to be comprised of exclusively conventional attitude items (i.e., verbal behavior in the form of expressions of appreciation toward an attitude object such as “I like X”; see, e.g., Eagly & Chaiken, 1993). Rather, other verbal acts (e.g., self-reports of behavioral engagement) or observed overt acts can serve as well. In fact, Byrka and Kaiser (2013) confirmed that traditional attitude items in the form of verbal expressions of appreciation, along with straightforward self-reports of attitude-relevant behaviors, can be represented by a single dimension of evaluative reactions to an attitude object (one’s own health in their example) because they represent a person-invariant ordered set of indicator items (see also Brügger, Kaiser, & Roczen, 2011, and Kaiser et al., 2018, for similar results regarding attitude toward nature and attitude toward environmental protection, respectively).

Many attitude measures that apply a Rasch-type model for the measurement of individual attitudes already exist in the literature (see, e.g., Howard, Ehrich, & Walton, 2014; Papanastasiou & Schumacker, 2014; Rojas-Tejada, Lozano-Rojas, Navas-Luque, & Pérez-Moreno, 2011). These measures are typically justified exclusively on the basis of the psychometric advantages of the Rasch model (e.g., specific objectivity – see, e.g., Rasch, 1977 – which implies certain aspects of sample-freeness; for a detailed example and explanation, see Kaiser et al., 2018). In other words, it is assumed that the measurement model can be chosen at will, without attending to the concept to be measured and by ignoring a latent attitude’s presumed theoretical link to its manifestations.

The Campbell Paradigm, by contrast, represents a measurement model that is grounded in an explicit conceptual model (a) of the expected relations between the attitude and various manifestations – the tripartite model of attitudes – and (b) of the specific connection between each single manifestation and a corresponding attitude – the Rasch model. Regarding the latter, the Campbell Paradigm thus speaks of a generic compensatory relation between a person’s attitude and the costs that are involved in engaging in a behavior (see, e.g., Kaiser, Arnold, & Otto, 2014; Taube et al., 2018).

In contrast to non-Campbellian attitude measures that apply Rasch-type models for the measurement of an individual attitude (e.g., plagiarism attitudes; see Howard et al., 2014), attitude measurement with the Campbell Paradigm thus involves an explicit exploration of the correspondence between the conceptually anticipated and the empirically identified item order. This occurs when, for example, we corroborate the idea that self-reporting one’s compliance is comparatively more demanding on average than verbally asserting compliance or verbally expressing approval (see, e.g., Byrka & Kaiser, 2013; Kaiser et al., 2010). Specifically, the Campbell Paradigm requires that a conceptually grounded ordering of the items operationally defines the latent attitude, which in turn must be (approximately) empirically confirmed by the Rasch-model estimates of the ordering of the indicator items by cost. Wilson (2005) identified this formal requirement as validity of the internal structure.

#### Conceptually Meaningful Item Order

Defining an attitude within the Campbell Paradigm involves carving out an assemblage of behavioral indicators and ordering them in terms of their costs. This set of indicators is thought to represent the behaviors people engage in when implementing their personal levels of a particular attitude (e.g., toward environmental protection), or stated somewhat differently, when pursuing a personal attitudinal goal (e.g., protecting the environment; see Kaiser et al., 2010). Depending on their personal levels of environmental attitude, people can wash their laundry in an energy-efficient way, vote for green political parties, and admit a certain degree of environmental concern on surveys.

Whereas certain behavioral indicators (e.g., glass and paper recycling) turn out to be rather undemanding – especially as self-reports (see, e.g., Geller, 1981) – others (e.g., taking public transportation or riding a bicycle to work or school) will be relatively more demanding but not as challenging as refraining from owning a car altogether (see, e.g., Kaiser, Midden, & Cervinka, 2008).

In the pursuit of a specific attitudinal goal, people are expected to deliberately and rationally choose from among several behavioral means that can help them cost-effectively realize their personal attitudinal goals (e.g., Kaiser et al., 2010). Thus, people favor relatively more convenient, socially accepted, and undemanding behaviors over more strenuous, socially proscribed, and demanding behaviors when manifesting their personal levels of a certain attitude. Consequently, the order of the behavioral responses that individuals use to implement their personal levels of a particular attitude operationally defines the conceptual understanding of the specific attitude under scrutiny. In reverse, the order of the behavioral indicators of a particular attitude can thus be used to validate a newly developed measure (see, e.g., Kaiser, 1998).

So far, the Campbell Paradigm has been used to develop Rasch-model-based attitude measures in various content domains: attitudes toward environmental protection (i.e., environmental attitude), nature (see, e.g., Kaiser, Brügger, Hartig, Bogner, & Gutscher, 2014; Kaiser et al., 2013), nature-preservation-related restrictions (see Byrka et al., 2017), global climate change (see Urban, 2016), health (see Byrka & Kaiser, 2013), mental vigor (see Beute, Kaiser, Haans, & de Kort, 2017), and social interaction (see Haans, Kaiser, & de Kort, 2007).

For illustrative purposes and to demonstrate its generic potential for attitude measurement beyond the known application domains, we will now apply the Campbell Paradigm to an arbitrary example (i.e., the 1984 US presidential election data) that was recently presented in a *Psychological Review* article in which the authors asked researchers to abandon the tripartite model as the measurement model for (explicit) attitudes (see Dalege et al., 2016). Note that this is an illustrative example. Thus, we do not expect *a conceptually grounded ordering of the items*, which is usually required in applications of the Campbell Paradigm. Still, even with such a suboptimally fitting example, the Campbell Paradigm can nontrivially, parsimoniously, and substantially account for people’s voting intentions and behaviors, something Dalege et al. did not even attempt to do.

## The Campbell Paradigm Applied to the 1984 US Presidential Election Data

We implemented three goals in this section. First, we tested our prime hypothesis derived from the Campbell Paradigm by investigating whether the 44 evaluative reactions to the two presidential candidates (i.e., Ronald Reagan and Walter Mondale) could form a person-invariant ordered set of items that could in fact be used as the basis for a Campbellian measure of pro-Reagan-anti-Mondale attitude. The resulting one-dimensional Rasch scale represents respondents’ joint expression of their appreciation for Ronald Reagan and their scorn for Walter Mondale.

Second, we tested whether a single attitude dimension could sufficiently represent all 44 evaluative reactions. Rather than corroborating only the theoretically anticipated dimension, we contrasted this one-dimensional Rasch model with two- and three-dimensional Rasch models to explore whether the information in the 44 items could be better represented by a more complex attitudinal model.

Third, we examined the predictive validity of the attitudinal measure that we developed. Here, we accounted for people’s intentions to vote for one of the two presidential candidates prior to the election and predicted their reports of how they had voted after the election in 1984. Note that, especially with measures at different levels of aggregation, such as in this case where attitude is measured with a multi-item scale and behavior is measured with a single item, expectations for comprehensive behavioral explanations (i.e., large effect size) are typically not high (see, e.g., Ajzen & Fishbein, 2005). In contemporary attitude research, for optimal correspondence and comprehensive explanations of behavior, attitude measures and behavior measures are typically required to be on the same level of measurement – either both single-item measures or both scales. This principle of aggregation has a long-standing tradition in attitude research (see, e.g., Eagly & Chaiken, 1993). On the basis of the Campbell Paradigm, however, we would expect an already fairly comprehensive explanation of people’s voting intentions prior to the election and a similarly comprehensive prediction of people’s self-reported voting after the election.

### Pro-Reagan-Anti-Mondale Attitude Within the Campbell Paradigm

Each respondent could express his or her appreciation for each of the two presidential candidates by means of 44 verbal behaviors: 15 evaluative statements and seven feelings related to each of the given candidates and their actions. The most straightforward application of the Campbell Paradigm with these 44 items consists of a bipolar attitude measure that ranges from a pro-Reagan-anti-Mondale sentiment on one end to a pro-Mondale-anti-Reagan sentiment on the other end of the scale. Such an attitude measure jointly captures a favoring of Reagan and a rejection of Mondale as a single latent behavioral propensity. In other words, it reflects how a person appreciates Ronald Reagan *and* simultaneously holds an unfavorable view of Walter Mondale. Note that the directionality of the scale is arbitrary. A pro-Mondale-anti-Reagan attitude would, of course, be formally equivalent. Note also that our example scale includes only cognitive and affective indicators but no behavioral indicators.

Operational Rasch scales require that the indicator items from which a person’s attitude level (in our case, the pro-Reagan-anti-Mondale attitude) is derived represent a single, person-invariant, and ordered Rasch-homogenous set of items. This test corresponds to the question of whether the Rasch model fits the data reasonably well. Moreover, the extent of a person’s attitude is derived on the basis of maximum likelihood estimation, which is the conventional way to score individuals with approaches that are based on the Rasch model (see, e.g., Embretson & Reise, 2000). The estimates represent logit scores. Logits stand for the natural logarithm of the favorable/unfavorable appraisal probability ratio across the entire response vector of a person. The smaller a logit value, the lower the particular person’s attitude, in this case, the less likely a person will be to express appreciation for Reagan.

Because our sample of 2,257 respondents was relatively large, we did not test for the statistical significance of the mean square (*MS*) statistic in assessing item fit but used the effect size interpretation instead (see Wilson, 2005). The *MS* statistic reflects the relative discrepancy in variation between the Rasch model’s predictions and the observed data, either for individuals or for items. An *MS* value of 0.75 corresponds to 25% less than the expected amount of variation, and an *MS* value of 1.33 indicates 33% more variation in the data than what was predicted by the measurement model. *MS* values in this range are regarded as a sensible threshold for instruments used in the scientific exploration of empirical relations (cf. Wright, Linacre, Gustafson, & Martin-Löf, 1994).

Our Rasch model test by and large revealed a reasonable statistical fit for our pro-Reagan-anti-Mondale Attitude scale. The *MS* fit statistics for the 44 indicator items in our scale all fell between an *MS* of 0.79 and an *MS* of 1.26. The traditional person reliability was replaced by the separation reliability (Wright & Masters, 1982), which reflects an estimate of the percentage of true person variance in the measure, which, in this case was rel = .92 (see Table 1).

**Table 1:** Exploration of the dimensionality of the attitude-toward-the-two-candidates items

Model	*G*^2^	npar	*r*_D1-D2_	rel_D1_	rel_D2_	rel_D3_
1D: pro-R-anti-M	90,311.86	45	–	.92	–	–
2D: pro-R & pro-M	83,032.13	47	−.44	.91	.88	–
3D: 2 D & Feeling	87,555.12	53	−.31	.87	.79	.64
*Notes*. *G*^2^ stands for the model fit statistic (its deviance), which is a log-likelihood statistic multiplied by −2 (see Adams, Wilson, & Wang, 1997). Differences in deviances are generally assumed to have a χ^2^-distribution with the difference in the number of parameters (npar) as degrees of freedom. Thus, we can statistically compare model fits of, for example, the less restricted two-dimensional (2D) model with the simpler one-dimensional (1D) model. Note that superior fit shows in smaller deviances. *r*_D1-D2_ = correlation between Dimension 1 and Dimension 2; rel = reliability estimates for Dimensions 1, 2, or 3. The correlations are estimates that have been disattenuated for measurement error (for technical details, see Adams et al., 1998).

### Pro-Reagan and Pro-Mondale Attitudes

Although our first Rasch model test revealed that the 44 statements about the candidates could be modeled as the expected pro-Reagan-anti-Mondale attitude within the Campbell Paradigm, we had yet to determine whether this single attitude offered a sufficient account of the electorate’s attitude toward the two candidates as captured by the 44 items. In other words, could these 44 items represent multiple attitudes toward the two presidential candidates?

Using the multidimensional random coefficients multinomial logit (MRCML) model (Adams et al., 1997) – a Rasch-type model that allows multiple person dimensions to be modeled simultaneously – we compared models describing a one-, a two-, and a three-dimensional attitude space. Like confirmatory factor analysis, the MRCML model allowed us to test a specific, predicted item-factor structure. In the present case, each item was assigned to only one dimension. In other words, multidimensionality was modeled as existing solely on the level of the concept and not on the item level, as is the case with simple structure in a factor analysis.

Whereas the one-dimensional model used all 44 items to reflect a single attitude toward the two candidates (i.e., a pro-Reagan-anti-Mondale attitude), the two-dimensional model used 22 indicators (for each candidate) to reflect each of two distinct attitudes: one pro-Reagan and one pro-Mondale. By contrast, our three-dimensional model separated both the pro-Reagan the pro-Mondale attitudes – solely represented by the 15 evaluative statements about each of the two candidates – from a “feelings dimension” (i.e., consenting to positive feelings and rejecting negative feelings). The feeling items were treated as a separate dimension under the assumption that they reflected some generic response tendencies (e.g., negative affectivity, a known mood-related bias in health research; e.g., Watson & Pennebaker, 1989), not least of all because the feeling items that were employed represented atypical attitude indicators.

Model fit was estimated with the *G*^2^ statistic, which is roughly χ^2^-distributed (cf. Adams et al., 1997). As can be seen in Table 1, the overall fit of the two-dimensional model, *G*^2^(47) = 83,032, was significantly better than for the one-dimensional, *G*^2^(45) = 90,312, or three-dimensional models,^3^ *G*^2^(53) = 87,555, with an increase in model fit of Δ*G*^2^(2) = 7,280 (*p* < .001) and Δ*G*^2^(6) = 4,523 (*p* < .001), respectively.

A two-dimensional model that spoke of two separate attitudes (i.e., one toward each of the two candidates) was also implied by the only moderate correlation between the two attitudes, which was *r* = −.44 (disattenuated for measurement error). On the one hand, this correlation corroborated the discriminant validity of the two attitude measures, and on the other hand, it indicated that a person who valued Reagan was simultaneously somewhat inclined to denigrate Mondale and vice versa. Separating the two attitudes did not necessarily have much relevance though, unless it could expand our understanding of people’s voting behavior.

### Explaining the Intention to Vote and Predicting Self-Reported Voting

**Figure 2:**
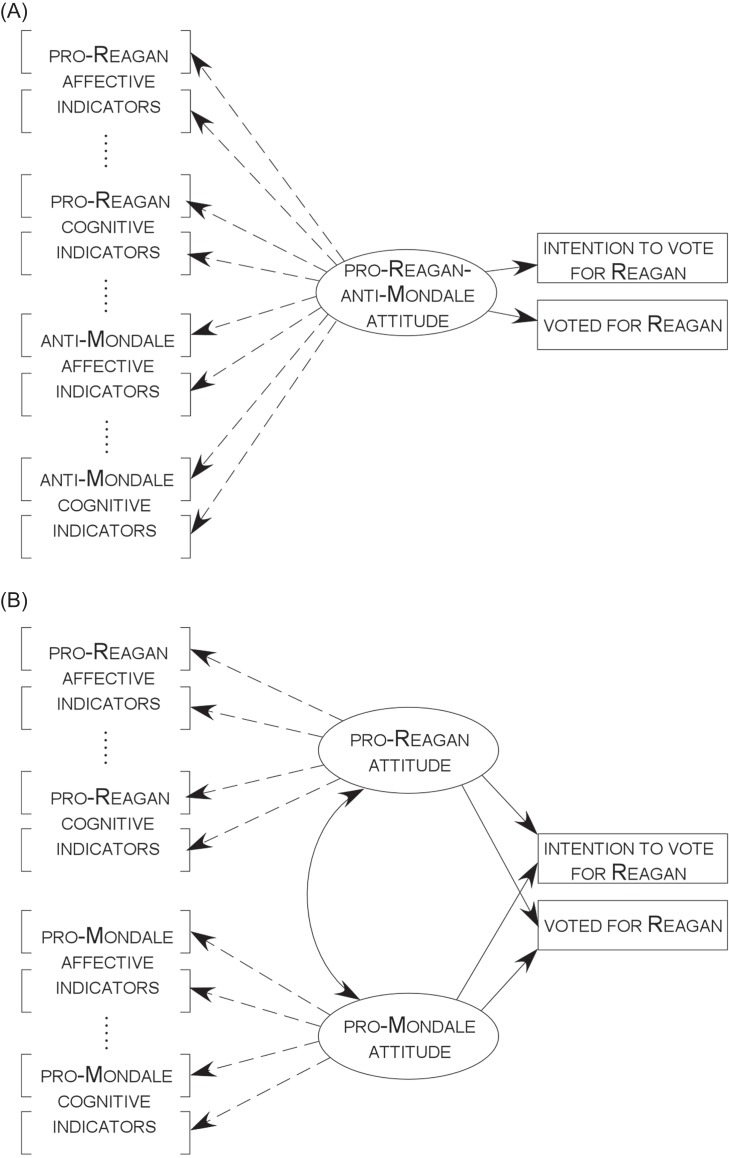
Depictions of two possible measurement/predictive models of attitudes toward the two candidates in the 1984 US presidential election. The number of affective and cognitive indicators (i.e., items) in this figure is lower than in the actual analyses for ease of presentation. The behavioral consequences of an attitude in this case are (i) the retrospective report of one’s actual vote for Reagan and (ii) the prospectively expressed intention to vote for Reagan. Dashed arrows specify indication. Solid arrows specify prediction. The double-headed arrow represents a correlation. (A) One-dimensional model; (B) Two-dimensional model.

In order to check whether attitude did indeed have an influence on a person’s intention to vote and on self-reported voting behavior, we used Mplus (Muthén & Muthén, 2011) to estimate a more complex model. Specifically, we estimated the models shown in Figures 2A and 2B, in which we simultaneously estimated regressions of two binary outcomes on the latent variable(s). These models, which combine measurement and prediction, allowed us to account for measurement error in the latent variables.

First, we discuss the results of the two-dimensional attitude model (Figure 2B). We used each person’s pro-Reagan and pro-Mondale attitudes to explain his or her intention to vote for Ronald Reagan (1) or Walter Mondale (0) before the election (*N* = 1,950) and to predict his or her self-reported actual vote for Ronald Reagan (1) or Walter Mondale (0) after the election (*N* = 1,376).

Jointly, the pro-Reagan and pro-Mondale attitudes accounted for 78.4% (Nagelkerke *R*^2^) of voting intentions and 74.5% (Nagelkerke *R*^2^) of actual voting behavior. Knowing people’s attitudes toward the two candidates raised the probability of correctly recognizing individuals with an intention to vote for Reagan from *p* = .58 to *p* = .90 and for correctly discriminating a Reagan from a Mondale voter from *p* = .58 to *p* = .88. The base rate (*p* = .58) was obviously already slightly in favor of Reagan in the surveyed sample.

For every logit increase in a person’s *pro-Reagan* attitude, the odds of expressing the intention to vote for Reagan increased by a factor of 14 (*p* < .001). For every logit increase in a person’s *pro-Reagan* attitude, the odds of actually voting for Reagan increased by a factor of 6.4 (*p* < .001).

For every logit increase in a person’s *pro-Mondale* attitude, the odds of expressing the intention to vote for Reagan decreased by a factor of 7.7 (*p* < .001). For every logit increase in a person’s *pro-Mondale* score, the odds of actually voting for Reagan decreased by a factor of 4.2 (*p* < .001).

Those who were above the mean on the *pro-Reagan* dimension (compared to those who were below the mean) were 4.6 times more likely to express their intention to vote for Reagan rather than Mondale (*p* < .001). They were also 4.3 times more likely (again compared to those who were below the mean) to actually vote for Reagan rather than Mondale (*p* < .001).

Those who were above the mean on the *pro-Mondale* dimension (compared to those below the mean) were about 5.7 times more likely to express their intention to vote for Mondale rather than Reagan (*p* < .001). They were also 5.5 times more likely (again compared to those below the mean) to actually vote for Mondale rather than Reagan (*p* < .001).

Now, consider the model in Figure 2A. The findings just reported deteriorated marginally when the pro-Reagan-anti-Mondale attitude – instead of the separate pro-Reagan and pro-Mondale attitudes – was used as the sole predictor in the two regression analyses. The pro-Reagan-anti-Mondale attitude alone accounted for 77.2% (Nagelkerke *R*^2^) of voting intentions and for 72.0% (Nagelkerke *R*^2^) of actual voting behavior. Knowing people’s attitudes toward the two candidates raised the probability of correctly recognizing individuals with an intention to vote for Reagan from *p* = .58 to *p* = .90 and for correctly discriminating a Reagan from a Mondale voter from *p* = .58 to *p* = .87.

For every logit increase in a person’s *pro-Reagan-anti-Mondale* attitude, the odds of expressing the intention to vote for Reagan increased by a factor of 290 (*p* < .001). For every logit increase in a person’s *pro-Reagan-anti-Mondale* score, the odds of actually voting for Reagan increased by a factor of 52 (*p* < .001).

Those who were above the mean on the unidimensional *pro-Reagan-anti-Mondale* attitude (compared to those who were below the mean) were 6.7 times more likely to express their intention to vote for Reagan rather than Mondale (*p* < .001). They were also about 6.3 times more likely (again compared to those below the mean) to actually vote for Reagan rather than Mondale (*p* < .001).

**Figure 3:**
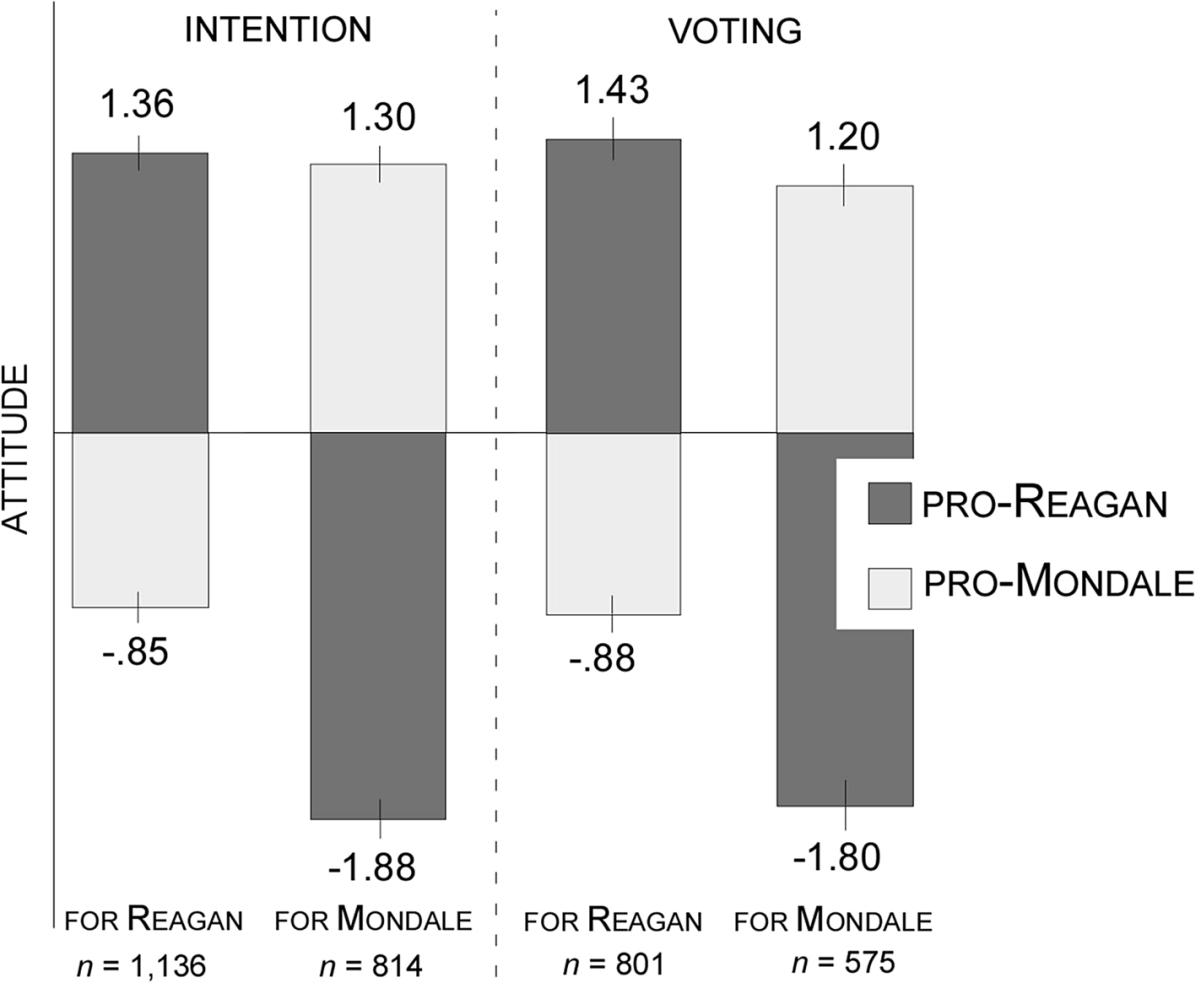
Mean voter attitude, measured as either pro-Reagan or pro-Mondale attitudes, of people who intended to vote and actually voted for either Reagan or Mondale in the 1984 presidential election.

Whereas the quantitative gains relative to the one-dimensional view might not be sufficient to justify the more sophisticated, two-dimensional view of people’s attitudes toward the two presidential candidates, the explanation for why someone chose to vote for Reagan or Mondale might. As expected, Reagan voters had positive views of their candidate and negative views of the challenger, and Mondale voters had positive views of their candidate and negative views of Reagan, as shown in Figure 3. This figure shows that Reagan and Mondale voters had comparable – in magnitude – positive attitudes toward the preferred candidate, but Mondale voters were significantly more negative than Reagan voters about the opponent. Also, consistent with Figure 3, the two sets of performance criteria – intention to vote prior to the election and self-reported voting after the election – were substantially correlated (ϕ = .91, *N* = 1,269). Even the means for the two attitudes for people who expressed an intention to vote for or who reported voting for either Reagan or Mondale were not significantly different and were thus comparable, which can be seen in the overlapping 95% confidence intervals in Figure 3 (see Cumming & Finch, 2005). Thus, in the following, we focused exclusively on people’s post-election self-reported voting, predicted by the attitudes toward the two candidates assessed prior to the election.

Measuring a single pro-Reagan-anti-Mondale attitude would have obscured the fact that people based their decision about which of the two candidates to vote for on two relatively distinct but correlated (*r* = −.44; see Table 1) reasons. Some chose Reagan because they valued him, others because they disliked the challenger (i.e., Mondale), and still others due to a combination of pro-Reagan and anti-Mondale sentiments. A one-dimensional, bipolar pro-Reagan-anti-Mondale attitude, by contrast, would have suggested that the electorate formed only one single attitude toward the two candidates in which a favorable view of one candidate was automatically counterbalanced by an unfavorable view of the other. For example, if appreciation for Reagan had increased, people’s valuation of Mondale would automatically have been lowered by the same amount. However, this compensatory view, which is consistent with a bipolar view of people’s attitudes, was compromised by the less-than-perfect negative correlation between the two attitudes.

In other words, a single bipolar pro-Reagan-anti-Mondale attitude would have implied that people tended to form one single view of both candidates jointly in which a favorable view of one candidate was compensated for by an unfavorable view of the other. In this case, people would obviously not have formed two more or less separate attitudes toward the two candidates. A bipolar pro-Reagan-anti-Mondale attitude, in contrast to two separate pro-Reagan and pro-Mondale attitudes, would also have implied that a presidential campaign could theoretically be exclusively positive about one candidate *or* exclusively negative about his opponent. There was nothing to gain from being positive about Reagan and negative about his opponent, Mondale. Both would at most result in a quantitative shift toward the pro-Reagan-anti-Mondale end and away from the pro-Mondale-anti-Reagan end of the scale. But with two imperfectly correlated attitudes, we can and must tell a different story.

The finding of two imperfectly correlated attitudes is consistent with the idea that presidential campaigns are won not only by stressing the positive aspects of one’s own candidate but also by stressing the flaws of the candidate’s opponent. This is, because some people seem to respond to the former and others to the latter. These two strategies for influencing the electorate correspond with people’s experiences with presidential election campaigns. A two-dimensional attitude model reflects these two strategies and presents them as reasonable.

## Conclusion

Following the lead of researchers such as Krantz, Luce, Suppes, and Tversky (1971), Michell (1999), and Rasch (1960/1980, 1977), we believe one key to a successful empirical science of behavior is the proper measurement of its core constructs. In this article, we demonstrated that the Campbell Paradigm – and, thus, its two parameters: the costs of implementing a particular behavior and the extent of an individual’s attitude – can be applied to Dalege et al.’s (2016) data to measure people’s pro-Reagan and anti-Mondale attitudes. Even in the context of Reagan versus Mondale, item order is apparently informative. The more pronounced a person’s pro-Reagan-anti-Mondale attitude, the more behavioral costs (in the form of, e.g., risk of social depreciation) the person will accept in order to endorse Ronald Reagan or reject Walter Mondale.

This is not to say that our specific results represent a matter that is free from controversy. It remains arguable whether political attitudes toward presidential candidates should be conceptualized as a system of two oblique attitudes or as a single bipolar attitude. From a technical point of view, two distinct attitudes (i.e., a pro-Reagan attitude and a pro-Mondale attitude) were barely superior to a single bipolar pro-Reagan-anti-Mondale attitude in accounting for people’s voting intentions and self-reported voting.

As predicted, we found that quantitative knowledge about the extent to which a person was dedicated to Reagan-electing or Mondale-rejecting goals (their pro-Reagan-anti-Mondale attitudes) substantially increased the probability of approximately *p* = .60 to a probability of around *p* = .90 for predicting a person’s voting intentions and his or her self-reported past voting for Reagan.

As a measurement model, the tripartite model describes the link between a latent attitude and some manifest evaluative statements – affective, cognitive, and behavioral responses – toward an attitude object (see Figure 1B). It provides a parsimonious account of individual attitudes. As a latent variable measurement model grounded in item order, the Campbell Paradigm represents a highly restricted version of the tripartite model that can be empirically tested and can actually fail (see Wilson, 2013). However, the ultimate criterion for any theory in behavioral science is its ability to account for manifest behavior – beyond verbal behavior.

Thus, next to its use as a measurement model for individual attitudes, the Campbell Paradigm also provides a theoretical account of any attitude-relevant individual behavior (including verbal behavior on questionnaires; see Kaiser et al., 2010). As we argue, the *same* model used to establish an estimation of the latent attitude is expected to also theoretically account for other forms of attitude-relevant behavior (see Figure 1C). This is in obvious contrast to some traditional practices and notions in social psychology.

For example, in the theory of planned behavior (e.g., Ajzen & Fishbein, 2005), the model for the measurement of its concepts is a rational-choice-based expectancy-value model (see, e.g., Eagly & Chaiken, 1993) and is as such quite different from the planned behavior model that is used to explain behavior (see, e.g., Ajzen & Fishbein, 1980). In the theory of planned behavior (i.e., the behavior-explanation model), attitude is typically one of three factors reflecting behavioral intention and behavior (the others being perceived behavioral control and subjective norms), not to mention the fact that in the theory of planned behavior, the principle of aggregation is usually mandatory. This means that attitude and behavior must be measured on the same level of aggregation, either as general or as specific measures. In other words, predicting a specific, single-item measure of voting behavior with a general, multi-item measure of a person’s pro-Reagan-anti-Mondale attitude would be expected to fail (see, e.g., Ajzen & Fishbein, 2005).

As a theoretical account of individual behavior, the Campbell Paradigm anticipates two compensatorily effective determinants of any manifest verbal or nonverbal behavior: the costs of implementing (i.e., the difficulty of) a particular behavior and the extent of an individual’s attitude (Kaiser, Arnold, et al., 2014). Not surprisingly, Arnold (2017) and Kibbe (2017) corroborated the compensatory effectiveness of behavioral costs and of individuals’ attitudes for attitude-relevant manifest behavior that was not already used to measure the attitude in question (see also Arnold & Kaiser, 2018; Byrka et al., 2017; Kaiser & Byrka, 2015; Taube et al., 2018).

As a measurement model, the Campbell Paradigm formally describes the relation between a latent concept (e.g., individual attitude) and manifest behavior (including verbal behavior on a questionnaire). As a behavior-explanation model, the Campbell Paradigm uses the quantitative estimate (i.e., the measure) of the latent attitude to explain the occurrence of the behavior in question. Still, when measurement – linking manifest behavioral indicators with individual attitudes – and explanation – predicting behavior with a latent attitude (see Figure 1C) – are treated as two logically and practically separate tasks that involve distinct behaviors, there is no logical fallacy to be caught in (cf. e.g., De Houwer et al., 2013). In other words, the circularity problem is avoided by separating the to-be-explained behavior from the behavioral indicators of an attitude (see Figure 1C).

With our research, we implemented the Campbell Paradigm as a reinterpretation of the tripartite model with data that were previously employed by Dalege et al. (2016). Even though such data are typical in attitude research, they were somewhat suboptimal for the Campbell Paradigm because the cost order of the verbal responses to the attitudinal objects – the two presidential candidates – could have been wider (for better examples, see, Byrka & Kaiser, 2013; Kaiser et al., 2018). With data that are better suited to fit a less restrictive measurement model than the Campbell Paradigm (see Dalege et al., 2016), we meant to demonstrate that, solely by including behavioral costs – with the Campbell Paradigm as the measurement model – the tripartite model can overcome its claimed weakness and account for all sorts of behavior.

We believe that Kaiser et al.’s (2010) Campbell Paradigm represents a highly restricted, sensible, and workable version of the traditional tripartite model and, thus, of a latent variable measurement model for (explicit) attitudes. As such, it (a) does not propose unverifiable causality between an attitude and the corresponding evaluative reactions, and it (b) does not propose this between an attitude object and an attitude either (see Figure 1A). In addition, the Campbell Paradigm (c) allows all types of behavioral reactions to be included (not only verbal behavior) when individual attitudes are measured as a latent variable (see Figure 1B), and it (d) allows unambiguous nontrivial predictions of behavior to be made as in the case of self-reported voting behavior (see Figure 1C). Most remarkable and in contrast to the state of affairs in social psychology, the Campbell Paradigm has the potential to help researchers rediscover actual behavior as the target for the science of behavior (e.g., see Kaiser & Byrka, 2015; Taube et al., 2018).

## Footnotes

^1^ Note that the tripartite model could – in principle – just as well be understood as a latent attitude causing *manifest* behavior and two *latent* psychological modalities (i.e., cognition and affect). This latter variant differs from the version depicted in Figure 1 but does not affect our reasoning.

^2^ Behavior here refers to any manifest, unequivocally describable, verbal, or nonverbal activity that is observable either with a given scientific method of investigation (e.g., a mark on a survey or an achievement in an experiment) or in everyday mundane activities, such as attending soccer games or praising a particular presidential candidate.

^3^ Note that we actually fit four different three-dimensional models using different codings (either rejecting or accepting both positive and negative feelings, and two other variants), but the one shown had the best fit.

## Appendix

### Participants and Procedures

In the 1984 American National Election Study (ANES), the pre-election sample (*N* = 2,257; response rate: 72.1%) was randomly assigned to either face-to-face or telephone interviews. Of this pre-election sample, 1,989 respondents (response rate: 88.1%) were again surveyed in the post-election interview (again face-to-face or over the telephone). The pre-election interviews (averaging 76 min) were all conducted before the November 6 election (starting September 4). The post-election interviews (averaging 46 min) were all conducted before January 25, 1985 (starting November 7, 1984).

### Data and Items

The data were made available by the Inter-University Consortium for Political and Social Research and initially collected by the Center for Political Studies in the Institute of Social Research at the University of Michigan for the national election studies, under the overall direction of Warren E. Miller; Santa Traugott was director of studies in 1984. The data were collected under a grant from the National Science Foundation. Neither the collector of the original data nor the consortium bears any responsibility for the analyses or interpretations presented here.

There were 15 evaluative statements tapping beliefs and seven tapping feelings about each of the two presidential candidates, and these were all handled in the same way as in Dalege et al. (2016). Beliefs were assessed by asking questions such as “In your opinion, does the phrase *hard-working* describe the *candidate* extremely well, quite well, not too well, not well at all?” The term *candidate* was replaced by either *Ronald Reagan* or *Walter Mondale* (and thus, there were 30 belief questions altogether, half related to each candidate), and *hard-working* was one of 15 attributes used to describe the candidates. The other 14 attributes were: (1) *moral*, (2) *knowledgeable*, (3) *inspiring*, (4) *providing strong leadership*, (5) *decent*, (6) *compassionate*, (7) *commanding respect*, (8) *intelligent*, (9) *kind*, (10) *setting a good example*, (11) *really caring about people like you*, (12) *understanding people like you*, (13) *fair*, and (14) *in touch with ordinary people*. Responses were coded 1 (= *not well at all*) to 4 (= *extremely well*). As Dalege et al. did, we also collapsed and recoded responses 1 and 2 to 0 (representing an unfavorable response) and 3 and 4 to 1 (representing a favorable response).

Feelings were assessed by asking “Has the *candidate* (because of the kind of person he is or because of something he has done) ever made you feel *angry*?” The term *candidate* was again replaced by either *Ronald Reagan* or *Walter Mondale* (and thus, there were 14 feeling questions altogether, half related to each candidate), and *angry* was one of seven feelings attributed to the candidate or his actions. The other six feelings were: (1) *hopeful*, (2) *afraid of him*, (3) *proud*, (4) *disgusted*, (5) *sympathetic toward him*, and (6) *uneasy*. Response options were *yes* and *no*. Whereas the *yes* responses to positive feelings (#1, #3, #5) were coded 1, the *no* responses were coded 0. The coding was reversed for negative feelings: *yes* responses were coded 0, and *no* responses were coded 1. In this way, 1 again reflected a favorable and 0 an unfavorable assessment.

In contrast to Dalege et al. (2016), there was no need for us to decide whether to apply a casewise or listwise deletion procedure or to decide how to deal with missing values. This is because the missing values could be accommodated by the Marginal Maximum Likelihood estimation procedure that we applied as the Rasch model estimation procedure used in the *ConQuest* software (Adams et al., 1998). We estimated individual attitude levels as “plausible values,”^4^ which allowed us to come up with a person score even when there was no variance in a person response vector (e.g., when all items were favorable or unfavorable). This is why we had attitude measures for all persons in the data set (*N* = 2,257), which was more than what Dalege et al. reported (*N* = 1,877 for attitude toward Ronald Reagan; *N* = 1,628 for attitude toward Walter Mondale). Note as well that we repeated all analyses described in this article with the 1,877 individuals who had complete data vectors for the Reagan-related reactions (i.e., containing no missing values) and the 1,628 individuals who had complete data vectors for the Mondale-related reactions. With this reduced dataset, we found basically the same results with no notable differences.

### Results

In Figure 3, the *y*-axis is the logit scale from the two-dimensional model, dimensions being pro-Reagan (dark gray) and pro-Mondale (light gray) attitudes. These two measures are in a common metric (after ensuring that items measuring these two attitudes have the same mean and variance).

The graph on the left (intention) represents the pro-Reagan (dark gray) and pro-Mondale (light gray) attitudes of those who expressed their intention to vote for Reagan (first bar from the left) and of those who intended to vote for Mondale (second bar from the left). Those who expressed their intention to vote for Reagan had a mean pro-Reagan attitude estimate of 1.36 and a mean pro-Mondale (rather an anti-Mondale) attitude estimate of −0.85. Those who expressed their intention to vote for Mondale had a mean pro-Reagan (again, rather an anti-Reagan) attitude estimate of −1.88 and a mean pro-Mondale attitude estimate of 1.30.

The graph on the right (voting) represents the pro-Reagan (dark gray) and pro-Mondale (light gray) attitudes of those who voted for Reagan (third bar from the left) and of those who voted for Mondale (fourth bar from the left). Those who voted for Reagan had a mean pro-Reagan attitude estimate of 1.43 and a mean anti-Mondale attitude estimate of −0.88. Those who voted for Mondale had a mean anti-Reagan attitude estimate of −1.80 and a mean pro-Mondale attitude estimate of 1.20.

Consider Figure 3 regarding intentions. We will interpret all four means (1.36, −0.85, 1.30, and −1.88) as the extent of or “intensities” in their respective attitudes. Statistically significant differences (at *p* = .01) between any two of these four means can be seen in nonoverlapping 95% confidence intervals (see Cumming & Finch, 2005).

Remarkably, those who intended to vote for Reagan had a pro-Reagan attitude that was higher in absolute (ignoring the sign) “intensity” (i.e., |1.36|) compared with the “intensity” of their anti-Mondale sentiment (i.e., |0.85|). By contrast, those who intended to vote for Mondale had a pro-Mondale attitude that was lower in “intensity” (i.e., |1.30|) compared with the “intensity” of their anti-Reagan sentiment (i.e., |1.88|).

The difference in relative “intensity” in the pro-Mondale and anti-Reagan attitudes of those who intended to vote for Mondale (1.30 − (−1.88) = 3.18) was much larger than the difference in “intensity” in the pro-Reagan and anti-Mondale attitudes of those who intended to vote for Reagan (1.36 − (−0.85) = 2.21). This pattern of findings about a person’s intention to vote for either Reagan or Mondale was fully reflected in the findings about voting for either Reagan or Mondale.

One interpretation of this might be that those who intended to vote (or voted) for Mondale might have expressed their intention or actually voted for Mondale not because of their appreciation for him but because of their extreme disdain for Reagan (because of their high anti-Reagan sentiment at −1.88 and −1.80). In turn, those who intended to vote (or voted) for Reagan might not have been too anti-Mondale because their anti-Mondale sentiment was substantially lower (at −0.85 and −0.88).
